# Perception of Polish pharmacy students on simulation exercise in pharmaceutical care for diabetes—a pilot study

**DOI:** 10.1186/s12909-024-05245-0

**Published:** 2024-03-14

**Authors:** Beata Plewka, Magdalena Waszyk-Nowaczyk, Magdalena Cerbin-Koczorowska, Piotr Przymuszała, Tomasz Osmałek

**Affiliations:** 1 Pharmacy Practice and Pharmaceutical Care Division, Chair and Department of Pharmaceutical Technology, Poznan University of Medical Sciences, 3 Rokietnicka Street, Poznan, 60-806 Poland; 2 Department of Medical Education, Poznan University of Medical Sciences, 7 Rokietnicka Street, Poznan, 60-806 Poland; 3 Edinburgh Medical School: Medical Education, University of Edinburgh, Chancellor’s Building, Edinburgh, EH16 4SB Scotland; 4 Chair and Department of Pharmaceutical Technology, Poznan University of Medical Sciences, 3 Rokietnicka Street, Poznan, 60-806 Poland

**Keywords:** Pharmacy students, Simulation, Self-efficacy, Pharmaceutical care

## Abstract

**Background:**

The transformation of a pharmacist’s role from that of a drug dispenser to an advisor and patient educator, partially accelerated by the COVID-19 pandemic, requires a thorough change in the pharmacy curriculum. Preparation for the provision of modern pharmaceutical services requires the use of the most advanced teaching methods, such as pharmaceutical simulation. Knowledge alone does not guarantee students’ readiness and motivation to take on new challenges in their professional work, but it seems crucial that graduates of medical faculties have the ability to practically apply their knowledge, including in new and nonstandard situations. Therefore, in our study, we proposed an intervention using a simulation method (peer role play) in teaching pharmaceutical care, and we assessed its impact on students’ levels of self-perceived confidence and self-efficacy in accordance with Bandura’s theory. The aim of the study was to verify whether the introduction of these types of classes could be a useful element of pharmacy curriculum renewal.

**Methods:**

The questionnaire-based study was conducted during pharmaceutical care peer role-playing classes in a simulation environment with a debriefing session among 85 final-year pharmacy students at Poznan University of Medical Sciences, Poland. The questionnaire consisted of two surveys: the General Self-Efficacy Scale (GSE) and a pre-post self-assessment and self-efficacy questionnaire.

**Results:**

There was a positive correlation between the GSE score and self-efficacy (*R* = 0.52, *p* < 0.0001). A statistically significant increase in the post-self-assessment of all the skills and competencies included in the survey in the field of pharmaceutical care of a patient with diabetes compared to the pre-values was also observed (*p* < 0.001). Additionally, the students’ self-efficacy in terms of communicating with patients was greater following the class than before the class (*p* < 0.001).

**Conclusions:**

The peer role-play active teaching method was found to be a cost-effective method allowing for an increase in the self-assessment and self-efficacy of pharmacy students in diabetic patient pharmaceutical care. However, further in-depth research is needed to fully confirm the effectiveness of simulation exercises for teaching pharmacy undergraduates.

**Supplementary Information:**

The online version contains supplementary material available at 10.1186/s12909-024-05245-0.

## Background

The image of the pharmacist, whose main task in the community pharmacy is to dispense the medicines prescribed by the doctor, is changing. The transformation of pharmacists’ roles from drug-oriented to patient-oriented specialists providing advanced services has been underway for several decades, but this transformation has certainly accelerated in many countries during the COVID-19 pandemic [1]. These changes result in the need for pharmacists to constantly monitor the surrounding reality, especially the health needs of societies, changes in the labor market, and the current state of knowledge [2, 3]. Therefore, there is much debate in the pharmaceutical community about the nature of what Hepler and Strand defined in 1990 as pharmaceutical care (PC) [4, 5]. Although several definitions have been proposed, all of them primarily relate to the optimization of pharmacotherapy [6, 7]. This is an important aspect of PC, but it is not the only one, as the duties of pharmacists in recent years have gained a much broader spectrum and include services such as vaccinations or disease screening elements, e.g., blood pressure measurements as well as providing health education to patients [8–11].

Even though pharmacists in previous studies expressed their readiness to expand the scope of pharmaceutical services, they often indicated a lack of appropriate practical preparation, which was lacking during their undergraduate pharmacy education, as well [11–13]. For instance, in a recent study carried out on Polish pharmacists, respondents revealed their willingness to serve as health educators despite obstacles such as inadequate qualifications and preparation during their undergraduate pharmacy education [11]. Similarly, research conducted among Polish students showed that they are aware of the role of the PC in community pharmacy practice but they would appreciate an increase in the availability of practical PC classes in the curriculum [14].

One of the important aspects of teaching pharmacy students also includes the previously mentioned changeability of the scope of the pharmacists’ professional tasks, which stems from a need to adapt to the current health needs of patients and market conditions of the pharmacies. Therefore, according to Maclellan [15], the essence of higher education should be to prepare students to generate new knowledge needed to solve future, yet unknown, problems. This is also at the core of Bandura’s theory of self-efficacy. Self-efficacy is an inner belief in one’s ability to cope with various situations, especially difficult ones [16]. Thus, a high sense of self-efficacy may be an internal motivator for acquiring knowledge, which, according to the assumptions of andragogy, is a main motivator in adults [17, 18].

The acquisition of knowledge and skills should not be two separate processes. Instead, there should be a transfer of knowledge into practice, enabling a broader understanding of a given aspect in accordance with the assumptions of conceptual learning theory [15]. Methods that not only incorporate the practical use of knowledge but also include reflection on the tasks performed are referred to as active learning methods [19]. In turn, simulation exercises are among the most advanced active learning methods and are especially useful for preparing students from medically oriented faculties for direct contact with a real patient [20]. According to Miller, knowledge (“knows”) is the basis from which the student moves to higher levels: competence (“knows how”), performance (“shows how”), and “does,” i.e., in the workplace setting [21–23]. In turn, simulation is a teaching method that combines knowledge and skills with attitudes, thus corresponding to all three domains of Bloom’s taxonomy and enabling us to reach the “shows how” level in Miller’s pyramid [24, 25]. For this reason, we assumed that allowing fifth-year pharmacy students to participate in classes using elements of simulation would positively affect their self-assessment in terms of skills and competencies as well as their self-efficacy beliefs.

As highlighted in the post-pandemic International Pharmaceutical Federation (FIP) report on global change in the pharmacy, one of the main challenges is introducing modifications in pharmaceutical education. It was also indicated that these changes should aim to increase students’ competencies and practical skills [26]. Therefore, in our opinion, it is necessary to identify teaching methods that will be engaging for students and, at the same time, allow them to acquire the skills necessary to practice as a pharmacist in a different setting than before. Thus, the aim of our study is to assess whether the educational solution we propose could be a useful element in modifying the pharmacy curriculum in alignment with challenges posed by the FIP and pharmacy students’ expectations.

For this purpose, we examined the impact of the peer role-playing method on students’ self-perceived confidence and self-efficacy in accordance with Bandura’s theory.

## Materials and methods

### Study design

The study was conducted using pre- and post-surveys, which provided qualitative and quantitative data, and was single-centered, cross-sectional, and interventional in nature. Consequently, we used both qualitative and quantitative methods to process the data.

The research was preceded by an analysis of students’ needs, which was the basis for the educational intervention [14]. The intervention consisted of preparing the peer role-play scenarios, which were then conducted in a simulated pharmacy room. Before and after the intervention, students completed questionnaires concerning their self-perceived confidence and self-efficacy.

### Study setting

The intervention was conducted during pharmaceutical care classes for fifth-year pharmacy students at Poznan University of Medical Sciences (PUMS) during the 2021/2022 academic year. The entire course consisted of five modules: asthma, hypertension, musculoskeletal disorders, type 1 diabetes, and type 2 diabetes. The study was conducted during the “Diabetes 2” module from December 2021 to January 2022. The duration of the classes was five lecture hours (1 lecture hour is equivalent to 45 min). They always took place after the students completed the “Diabetes 1” module, which introduced them to issues related to diabetes and familiarized them with relevant equipment: glucometers and insulin pens. The second part of the classes was aimed at learning how to use previously acquired knowledge and skills in pharmacy practice. Before our intervention, this portion of the classes consisted of performing exercises using medical equipment. During our intervention, this was conducted in the Medical Simulation Center and consisted of role-play scenarios that also included elements of providing patient education.

### The intervention

We designed three scenarios during which students played the roles of a pharmacist and a patient. The scenarios are presented in Table 1.

**Table 1:** The outline of the scenarios used in the study

Title of the scenario	Inclusion of basal insulin	Hypoglycemia	Alternative site testing (AST)
Patients	A married couple	The patient is not present in the pharmacy. His roommate seeks help in a nearby pharmacy and is in constant telephone contact with a third roommate who stayed with the patient.	A young woman with type 1 diabetes
Description	The patient tries to hide the fact that, in addition to regular medications, he received his first prescription for insulin because he does not see the need for injections. His wife is aware of this and tries to suggest it to the pharmacist.	A young man has hypoglycemia after an intense exercise session. His friends who were with him do not know about his illness and are looking for help at a nearby community pharmacy.	A patient with type 1 diabetes fills a prescription for glucometer strips and mentions to the pharmacist that she feels discomfort due to pain in her fingers after frequent pricking.
Pharmacist’s main tasks	Identifying the problem followed by convincing the patient that he should take insulin. Providing education on the use of the insulin pen.	Identifying that the patient’s condition was the result of hypoglycemia and instructing his colleagues on what actions they should take	Providing education on AST

**Fig. 1:**
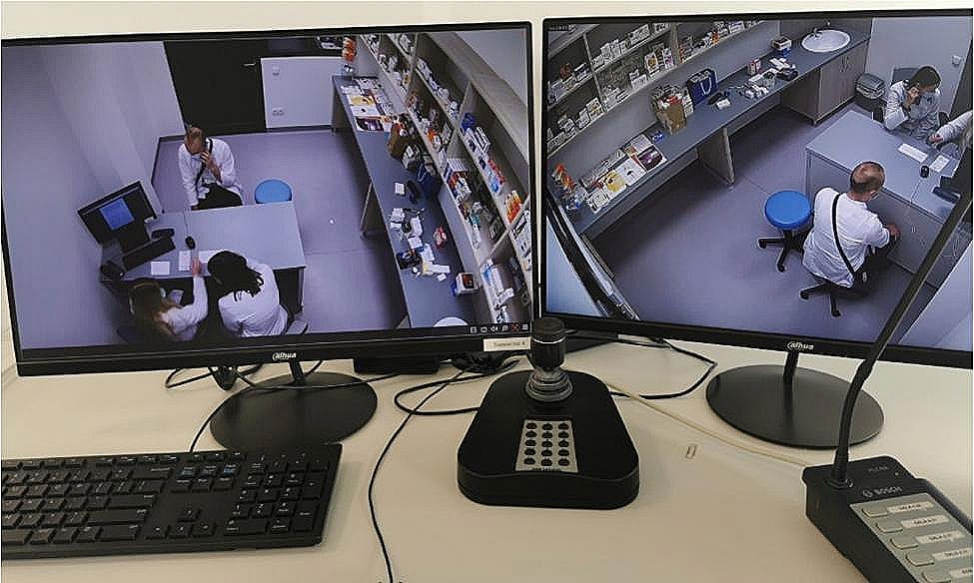
The view of the pharmacy during the simulation scenario role-play from the perspective of the teacher (photo by M. Waszyk-Nowaczyk)

**Fig. 2:**
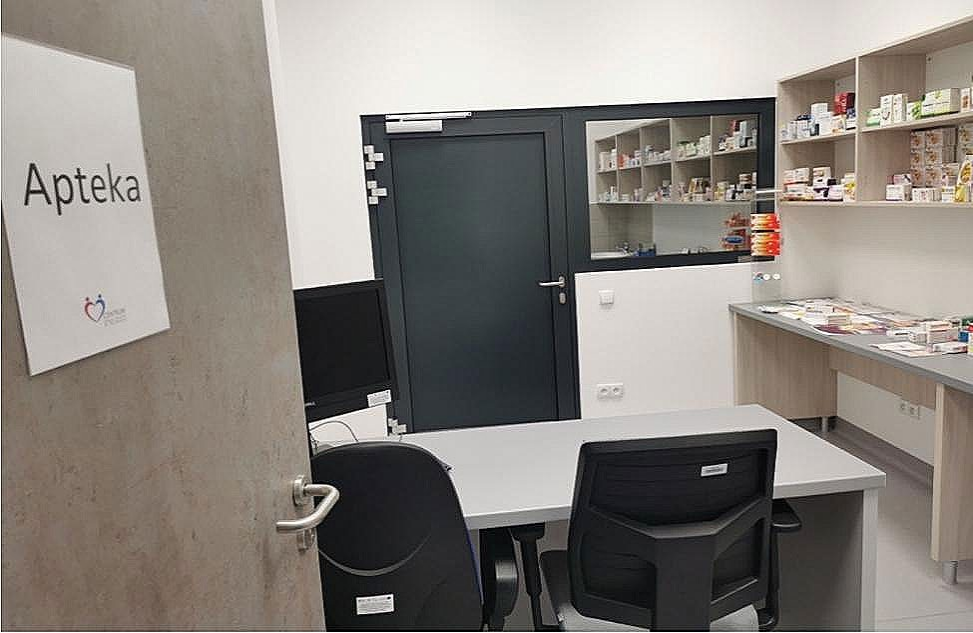
Simulated pharmacy room (photo by M. Waszyk-Nowaczyk)

**Fig. 3:**
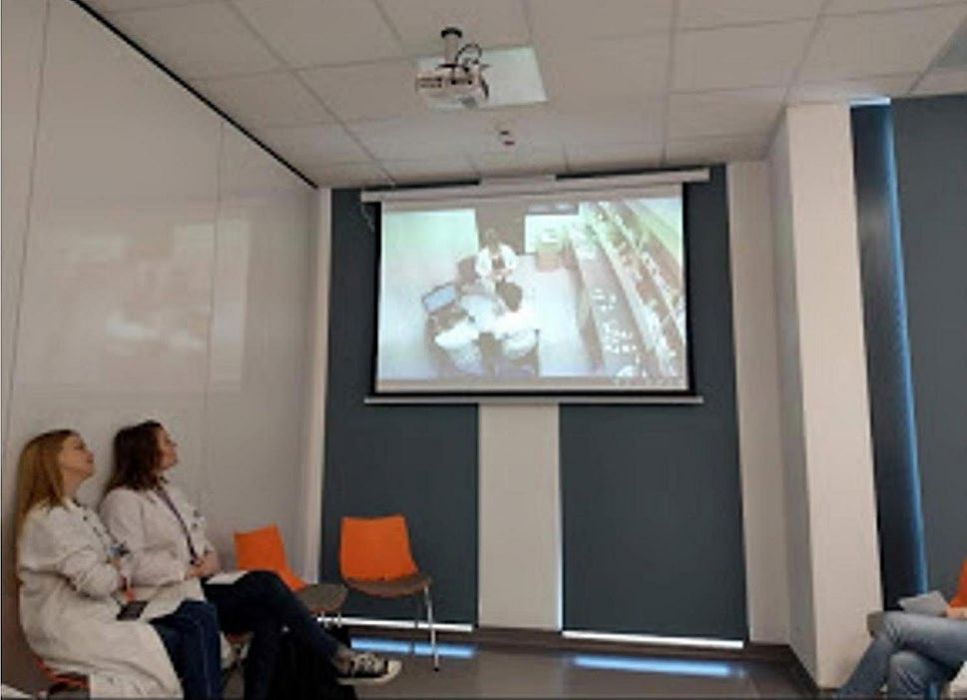
A debriefing room during a role-play scenario (photo by M. Waszyk-Nowaczyk)

The role-playing took place in a separate room, which was made to resemble a community pharmacy. Each scenario involved one student as a pharmacist, one or two students as a patient, and two observers who were also present in the pharmacy room but only passively observed the scenario with a focus on verbal and nonverbal aspects of communication. Students not directly involved in the scenario observed it on a screen in a debriefing room. Only students who played the role of patients were familiarized with the entirety of the scenario. There were between 8 and 10 students in each group, under the supervision of one teacher (Figs. 1, 2 and 3).

A different student took on the role of pharmacist for each scenario. In addition, each scenario was preceded by a short pre-briefing session, primarily to introduce students who played the role of the patient to the scenario. After each session, a debriefing session was conducted in accordance with the assumptions of the Pendleton model of providing feedback. First, they focused on the positive aspects that were indicated by the participants of the scene, the observers, and the teacher. Subsequently, in the same order, aspects that could have been performed differently were discussed [27].

While conducting the study, we followed the guidelines for reporting evidence-based practice educational interventions and teaching (GREET) checklist [28, 29]. A description of the intervention according to the GREET guidelines is provided in Table 2.

**Table 2:** The educational intervention checklist was created based on the GREET tool

Criterion	Description
Preparation	
Learning needs	These were identified in the previous survey research: perceived lack of PC classes and limited use of active learning methods [14]. In addition, the intervention set out to use these active learning methods to achieve the learning outcomes listed in the Regulation of the Minister of Science and Higher Education of July 26, 2019 [30], such as: E.U14. providing patient education related to the medications they use and other problems regarding their health and disease, and preparing personalized educational materials for the patient; E.U5. planning, organizing, and conducting pharmaceutical care; E.U6. conducting pharmaceutical consultations in the process of pharmaceutical care and pharmaceutical consulting.
Intervention development process	The scenarios used during the classes were developed by academic teachers, who are also practitioners working in a community pharmacy.
Theory	As the intervention involved adult learners, we adopted the assumptions of andragogy, according to which, in adults, internal motivators play a key role in the learning process. Thus, the acquired knowledge should be coherent, practical, and related to the adults’ work [31]. Therefore, the activities they participate in must be engaging and practice oriented. Furthermore, we used Bandura’s self-efficacy theory to design the surveys and interpret the results.
Intervention	
Educational strategy	The strategy involved simulation-based peer role-play in a community pharmacy-like setting. Classes included a pre-briefing, a scenario, and a debriefing.
Instructors	Two authors were also instructors during the intervention. They are both academic teachers with experience in simulation as well as practicing pharmacists.
Schedule and attendance	During the intervention, classes were conducted according to the previously adopted schedule. Students were divided into small groups of 8 to 10, and each group attended one class scheduled in the “Diabetes 2” module. Attendance was obligatory to receive course credit.
Content/subject and learning objectives	During classes, the issues of pharmaceutical care in diabetes were discussed. They focused on educating the patient in the area of practical skills, i.e., measuring glucose levels and administering insulin or glucagon, as well as dealing with hypo and hyperglycemia. Their aim was for students to acquire practical skills and competencies to play the role of a patient educator.
Materials	Students were provided with teaching aids, such as the latest guidelines in diabetic patient care and medical devices (glucometers, pens, etc.)
Incentives	No incentives were given to the learners. Participation in classes was mandatory, but participation in the study and completing the surveys was voluntary and anonymous.
Assessment	The level of students’ self-efficacy and skills self-assessment was evaluated. An assessment of knowledge was not planned due to logistical and time constraints.

Two questionnaires were used in the study: the General Self-Efficacy Scale (GSE) and a pre-post self-assessment questionnaire. The GSE is a 10-item scale assessing subjects’ beliefs about their ability to cope with difficult situations and life challenges. Self-efficacy refers to personal agency, i.e., the sense of self-efficacy defined by Bandura as a belief in the power of the impact of undertaken actions on achieving success. This concept was created in 1981 in Germany by Matthias Jerusalem and Ralf Schwarzer [32, 33]. The scale is available in 32 languages, including Polish. The authors of the Polish-language scale are Schwarzer, Jerusalem and Juczynski. It is available online, and the authors consent to its use in scientific research [33, 34]. The Polish scale was assessed for reliability and validity, obtaining a reliability score of 0.78 and an average Cronbach’s alpha of 0.85 [35]. For each question on the scale, there are four possible answers: yes, rather yes, rather no, and no. For each answer, points from 1 to 4 are awarded, with “no” being scored the lowest and “yes” the highest. Then, the obtained points are summed up, and the greater the value is, the greater the respondent’s sense of self-efficacy.

The second questionnaire was constructed by the first author on the basis of published data [36–39]. The data were then reviewed by a PC and medical education specialists. The final version consisted of three parts: self-assessment of skills, self-assessment of competence, and self-efficacy (Appendix 1). The first contained seven statements on the subject’s belief in their own PC skills, with a seven-point Likert scale ranging from “completely disagree” to “completely agree”. The second contained six statements assessing the subject’s competencies in specific aspects, with answers also being given on a 7-point Likert scale, from “very difficult” to “very easy.” In the third part, a visual analog scale (VAS) was used to test self-efficacy for the ability to communicate with the patient. Two statements were placed on either end of a 10 cm line: “lack of self-confidence in communication with the patient” and “highest possible self-confidence in communication with the patient.” The respondents marked the space between these two statements with a vertical line, thus indicating the level of their perceived ability to communicate with the patient. The aim was to distinguish general self-efficacy from self-efficacy in a specific situation of communication with a patient.

**Fig. 4:**
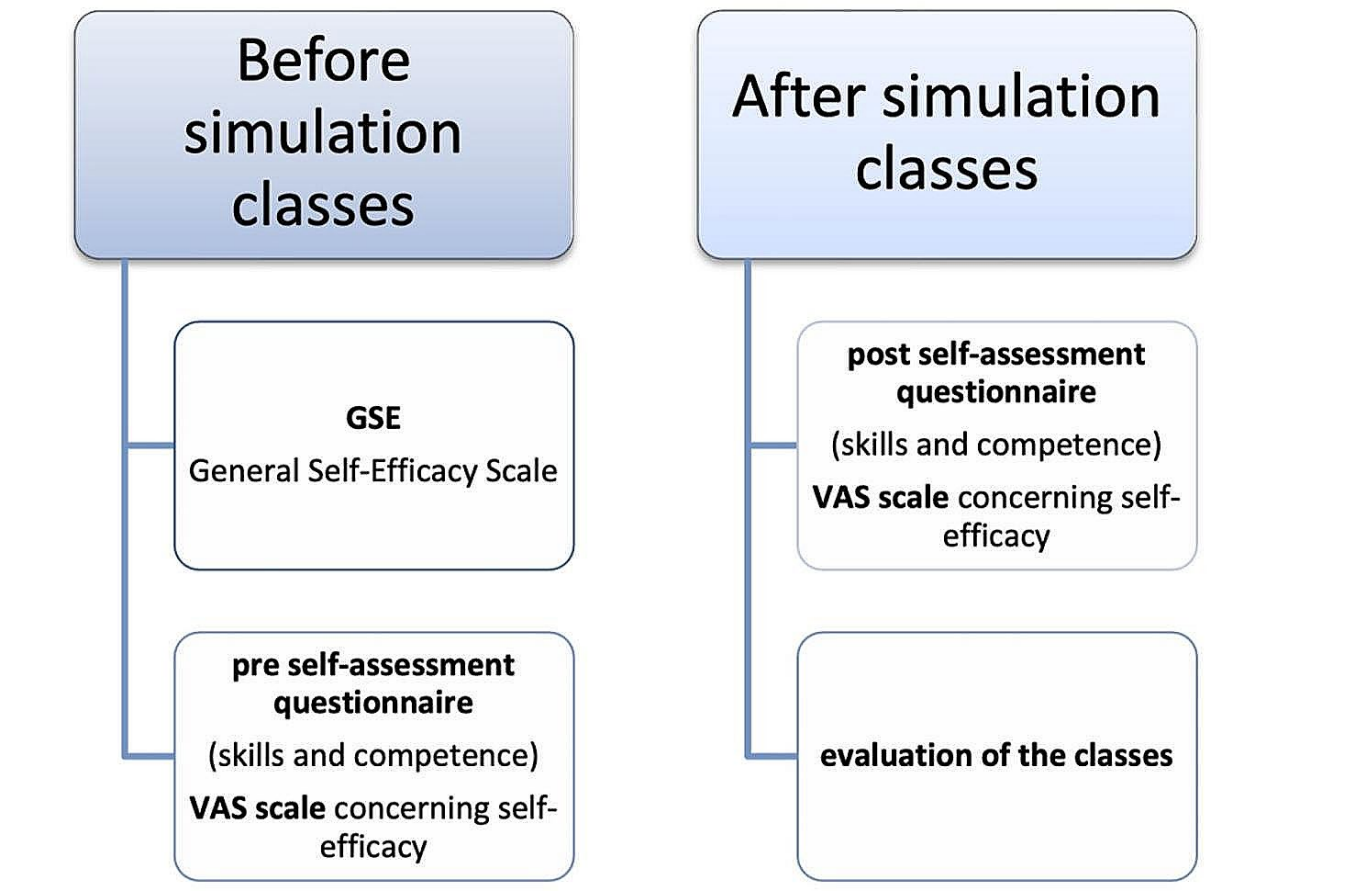
Types of surveys used before and after the intervention

At the end of the classes, students were also asked to answer two open-ended questions: “What did you like about the simulation classes?” and “What did you not like about the simulation classes?” The answers were provided in writing. The survey flowchart is shown in Fig. 4.

### Participants of the study

The participants in the study were fifth-year pharmacy students at the Poznan University of Medical Sciences (PUMS). The inclusion criteria were participation in the pharmaceutical care classes and voluntary consent to participate in the study. The exclusion criterion was a lack of consent to participate in the study.

### Data analysis

The study included two types of data: quantitative and qualitative. Quantitative data were statistically analyzed using the Mann‒Whitney U test and the Wilcoxon signed-rank test. Correlations were calculated using Spearman’s rank correlation coefficients. Statistica (version 13.3) (TIBCO Software, Inc., Palo Alto, USA) was used for the statistical calculations.

Qualitative data were analyzed by two independent researchers. We chose to follow Braun and Clarke’s recommendations for a thematic analysis of these data [40]. Guided by these outlines, researchers began by reviewing the data. Based on this, they generated initial codes. In the following stages, they searched for and reviewed the themes. Then, they defined and named the themes, and finally prepared a summary report.

### Ethical considerations

In accordance with the guidelines of the local Bioethics Committee, anonymous survey research does not require the committee’s approval. The study project was presented to the Bioethics Committee at Poznan University of Medical Sciences for its opinion, which confirmed that the study was not a medical experiment and that its approval was not necessary (decision from January 16, 2020). In addition, students were informed orally that participation in the survey was voluntary and anonymous, that they had the right to withdraw at any stage without giving a reason, and that by completing and returning the questionnaires, they consented to participate in the study. The results of the surveys and the students’ participation in the study did not affect the completion of the course or the grade obtained. We have made every effort to ensure that our research meets the standards set out in the Ethical Guidelines for Educational Research, a document adopted by the British Educational Research Association (BERA) in 2018 (fourth edition) [41].

## Results

The analysis of the answers obtained in the surveys involved verifying whether individual aspects have changed significantly after the intervention. Additionally, we assessed the correlation between students’ sense of self-efficacy and their level of self-perceived confidence in the pharmaceutical care of diabetes patients.

### Participant demographics

Eighty-five students participated in the study; 22 were men (18.7%), and 63 were women (81.3%). The age of the respondents ranged from 22 to 30 years (mean = 23.6 ± 1.15, median = 23, Q1 = 23, Q3 = 24). All of them were fifth-year pharmacy students, and 4 of them were also students of other faculties: physiotherapy (2 persons), cosmetic chemistry (1 person), and biology (1 person). More than half of the students (54 people, 63.5%) had previously participated in simulation exercises as part of the facultative course “Diagnostic tests in a community pharmacy.”

### General Self-Efficacy Scale

The average self-efficacy score for the entire group of respondents was 29.49 ± 3.64. On average, the score was approximately one point greater in men than in women, but the difference was not statistically significant (29.21 ± 3.76 vs. 30.32 ± 3.02, *p* = 0.1435). Although the observed scores were slightly greater, earlier participation in simulation exercises had no statistically significant impact on the average GSE score (29.74 ± 3.33 vs. 29.06 ± 4.03, *p* = 0.1430).

### Pre and post self-assessment questionnaire

The pre-post analysis revealed statistically significant differences in students’ self-assessment with regard to all aspects of competencies, skills, and self-efficacy following the simulation exercises compared to the pre-score (Table 3). Analysis of pre-intervention scores showed that male students initially assessed their competencies in the field of interpersonal communication during patient education better than women did (*p* = 0.0340). In addition, students who had previously participated in simulation exercises rated their knowledge of the use of glucometers higher than those who had not previously participated in such activities (*p* = 0.00001). They also felt more competent in educating patients (*p* = 0.0046).

In turn, an analysis of post-intervention results revealed that students who had already participated in simulation-based classes often presented greater self-assessments of their skills. The results were significantly greater than those of students who participated in a simulation for the first time in terms of the ability to communicate with the patient (*p* = 0.0046), the transfer of knowledge to the patient in an understandable way (*p* = 0.0001), and the ability to identify the patient’s health problems (*p* = 0.0102) and needs (*p* = 0.0084). The participants also reported that better knowledge (*p* = 0.0008) and competence (*p* = 0.0037) are necessary for providing patient education or that it is easier to instruct patients on specific skills (*p* = 0.0218). No other statistically significant differences in terms of student sex or previous participation in simulation exercises were observed.

**Table 3:** Students’ self-assessment ratings before and after the classes

Self-efficacy assessment on the VAS (Visual Analog Scale) on a 10 cm line where 0 = lack of self-efficacy in communicating with the patient and 10 = the highest possible self-efficacy in communication with the patient (*n* = 85)					
Self -efficacy		M	Q1	Q3	p Value^1^
	PRE	5	4	6	< 0.001
	POST	7	5	7	
Self-perceived confidence regarding skills on a seven-point Likert scale, where 1 = I completely disagree and 7 = I completely agree (*n* = 85).					
		M	Q1	Q3	p Value^1^
1. I am convinced that I can communicate effectively with the patient.	PRE	5	4	5	< 0.001
	POST	5	5	6	
2. I am convinced that I can convey knowledge to the patient in an understandable way and instruct them accordingly (e.g., how to use glucometers or pens).	PRE	5	4	5	< 0.001
	POST	5	5	6	
3. I have the appropriate skills in the field of interpersonal communication to conduct patient education.	PRE	5	4	5	< 0.001
	POST	5	5	6	
4. I have the appropriate skills in the field of interpersonal communication to conduct patient education.	PRE	5	4	6	< 0.001
	POST	5	5	6	
5. I can identify the patient’s health problems and respond to them accordingly.	PRE	5	4	5	< 0.001
	POST	5	5	6	
6. I can identify the patient’s health needs and respond to them accordingly.	PRE	5	4	5	< 0.001
	POST	5	5	6	
7. I have the appropriate knowledge to educate patients on the use of glucometers and pens.	PRE	4	4	5	< 0.001
	POST	5	5	6	
Self-perceived confidence regarding competencies on a seven-point Likert scale, where 1 = very difficult and 7 = very easy					
		M	Q1	Q3	p Value^1^
1. Providing the patient with knowledge and instruction in the field of skills is…	PRE	3	3	4	< 0.001
	POST	5	4	5	
2. Interpersonal communication is…	PRE	4	3	5	0.011
	POST	5	4	5	
3. Reading nonverbal messages is…	PRE	5	3	5	< 0.001
	POST	5	4	5	
4. Identifying and responding to patient problems is…	PRE	4	3	5	< 0.001
	POST	5	4	5	
5. Identifying and responding to patient needs is…	PRE	4	3	5	< 0.001
	POST	5	4	5	
6. Educating the patient on the use of glucometers and pens is…	PRE	4	3	5	< 0.001
	POST	5	4	6	

^1^Data were analyzed using the Wilcoxon signed-rank test; p values are significant at the 0.05 level; M, median; Q1, lower quartile; and Q2, upper quartile

### Correlation analysis

Correlation analysis revealed positive correlations between students’ self-perception of confidence in the pre and post-survey scores and their self-efficacy and GSE scores. When correlated with the GSE score, the correlation strength was weak (below 0.4) in almost all cases. The only exception was the statement: “I am convinced that I can convey knowledge to the patient in an understandable way and instruct them in skills (e.g., how to use glucometers or pens),” where a moderate correlation was observed (R = 0.48, p < 0.0001). The results of the GSE and self-efficacy in communicating with the patient were also compared to determine whether the general sense of self-efficacy affects self-esteem in a specific situation. This relationship was positive and moderate in strength (R = 0.52, p < 0.0001).

### Evaluation of simulation classes

As a result of the analysis of qualitative data provided by students regarding the simulation classes, we generated four themes. The results are presented below, and a summary of the results is also given in Table 4, along with an indication of their elements related to the aim of the study and sample statements.

## It is good to know what could have been done better—without judgment

The first theme captures students’ impressions of the analysis of the scenario during the debriefing after completing the role-play. The aspect of assessment was highlighted. The title of the theme reflects the question asked during the debriefing—what could have been done better/differently? The idea behind the classes we conducted was to strengthen the good sides and build positive self-esteem in the participants and to express criticism in a constructive way without judgment. The students noted that a joint analysis of the behavior of the person acting as a pharmacist at the scene does not need to be stressful for them, and even if they make mistakes, they will be shown how to correct them. The intervention was therefore viewed as being potentially helpful to the participants, saving them from making similar mistakes in their professional work in the future.

*It was possible to make mistakes that were later corrected, and thanks to this, I know that I will not make them in the future.*

*It is good to receive constructive feedback.*

## All hands on deck!

The second theme pertains to the engagement of all participants during the classes. This is because, on the one hand, the simulations are interesting and dynamic, and on the other hand, the teacher creates space for everyone to express themselves. Each person is an active participant in the debriefing session. Additionally, topics related to professional work and the acquisition of practical skills increased students’ involvement. The students indicated that even if they did not personally participate in the study, they felt a sense of responsibility toward the patient and empathized with the role of the pharmacist. The participants also appreciated the opportunity to jointly search for the optimal solution to a given situation during the debriefing.

*Each person could present their point of view.*

*The scenarios were not obvious, and each of us had a different idea of what could be done.*

*While watching the scene, I felt like I was in a pharmacy serving a patient; I was looking for the best solutions in my head.*

## Just like in a real pharmacy

The third theme encompasses practice orientation in the classroom. The most important elements of this topic are the realistic nature of the scenario and the environment itself. The situations played out by the students closely reflected real-life scenarios, which allowed them to learn how to use their knowledge in practice. Additionally, they were required to think outside the box and to look for nonobvious solutions. Additionally, some students had never had contact with a real patient before, and they especially appreciated the opportunity to take part in a role-playing scenario.

*I had to figure out what was going on and then find a solution, just like in real life.*

*It was the first time that I had the opportunity to serve a patient in a pharmacy.*

*We should have more classes like this before post-graduation internships.*

## Stress

The last theme covers one of the disadvantages of the classes noticed by the students—stress. It accompanied many of them and was primarily related to the fear of being watched. Participating in a simulation scenario in a room with cameras while being observed by other students on-screen caused discomfort for some students. Some people were nervous about someone watching them, while others were nervous about the mere fact that a camera was present in the room. Some participants were afraid that they did not have enough knowledge or that they would make a mistake that they would not make under normal conditions.

*I do not like being watched and it stressed me out at the beginning.*

*The camera always makes me stressed.*

*The downside for me was that under stress you can sometimes forget basic things.*

**Table 4:** Themes generated during the study and their relationship to the aim of the study

Theme name	Sample statements	Meaning
It is good to know what could have been done better - without judgment	*“Pointing out positive sides - strengthening the belief that we have the skills.”* *“Good to hear what can be improved.”* *“No judging, discussing scenes without stress.”*	Conducting a debriefing based on the Pendleton model reassured the students that the classes were not evaluative in nature.
All hands on deck!	*“The classes were conducted in a very interesting way.”* *“Everyone had the opportunity to comment on the scene.”*	This form of classes enabled students to become active participants, i.e., strengthening their internal motivation to learn, in accordance with the assumptions of andragogy.
Just like in a real pharmacy	*“I felt like I was in a real pharmacy.”* *“The script was realistic, it raised problems that are difficult to solve only with theoretical knowledge after graduation.”* *“A scenario was similar to a possible situation in a pharmacy. It reflects reality well.”* *“I liked the opportunity to put theoretical knowledge into practice.”* *“I liked the possibility of using knowledge in a real situation.”*	The proposed scenarios used during simulation exercises and the skills acquired during the role-play and debriefing were assessed by the students as useful in the context of their future professional work, which meets the assumptions of the Kirkpatrick model. This model assumes that participants’ satisfaction is one of the basic elements of course evaluation [42].
Stress	*“The disadvantage of this type of class is the increased level of stress.”* *“The downside was the audience and the fact that someone was observing the course of the scene.”*	Despite pointing out the stress associated with role-playing, students in self-perceived confidence surveys indicated an increase in self-esteem, which allows us to assume that it did not demotivate them.

## Discussion

This study aimed to design an educational intervention and evaluate it in terms of students’ expectations as well as the impact of the simulation on their self-confidence in performing PC services. We also attempted to interpret the meaning of the simulation exercises in accordance with the assumptions of Bandura’s theory. Our analyses aimed to answer the question of whether the introduction of simulation classes could be an element of a revised pharmacy curriculum and an appropriate response to the students’ needs.

As the results of the pre-post surveys show, students benefited from the classes through an increase in their self-efficacy and self-assessment of their skills and competencies. Therefore, there is a good chance that they will implement the observed behavior in their professional work. However, it should be noted that the simulation in our study involved not only students actively participating in the scenarios but also observers—not everyone had the opportunity to participate actively in the scenario. As a recent study conducted on Polish medical students showed, students observing the scenario could also achieve significant improvements in their self-efficacy, although the improvements among students actively participating as doctors were greater for some specific skills [43]. The fact that observers also benefit from classes can be explained by the concept of sources of self-efficacy identified by Bandura: enactive mastery experiences, vicarious experiences, verbal persuasion, and other social influences as well as physiological and affective states [44]. Students who actively participate in scenarios mostly involve enactive mastery experiences, which correspond with the effects of one’s own successes and failures on their self-efficacy beliefs. However, students observing the scenario can also draw from it, using it as a point of reference and making judgments about their own abilities, thus forming their own vicarious experiences. An important element of our classes was the debriefing, during which students received extensive feedback—both from the teacher and peers. It has been proven that learning based on feedback is effective in the context of acquiring knowledge and self-efficacy [45, 46].

Taking mentioned above aspects into consideration, in our simulation classes, the increase in self-efficacy and self-assessment of skills and competencies was due to the creation of an environment in which students could learn both by observation and by giving and receiving feedback. Our intervention was based on an implementation of the simulation method within the pharmacy curriculum, as previous research has shown that this particular method significantly influences students’ self-perception of their skills and competencies [47–49]. In our study, this contributed to an improvement in students’ self-confidence in communication. A similar group of pharmacy students in terms of age and number participated in a study by James et al., which was also designed to determine the impact of simulation on the participants’ self-esteem [39]. As in our study, the authors reported an increase in confidence in conducting patient consultations with a simultaneous decrease in students’ perceived difficulty in conducting such consultations. However, to confirm the effect of simulation on students’ self-perceived confidence in aspects of PC, similar research should be carried out in a control group that participates in classes on similar topics but is conducted using a different, less advanced method. In our study, we decided not to implement two different educational solutions for ethical reasons because it would mean that some students would take classes that could be potentially less beneficial for them. Moreover, the university’s policy does not allow classes to be conducted using two different teaching methods, as it must be consistent with the previously adopted syllabus. 

According to the Kirkpatrick model, which assumes four levels of course effectiveness assessment, the appraisal should always start with the first level, which evaluates the participants’ reactions—their satisfaction, engagement, involvement in the learning experience, and relevance of new knowledge to their work [42]. Although a positive reaction does not guarantee the occurrence of learning, a negative reaction almost certainly reduces its possibility. The majority of students who were asked to express their opinion in our survey about the classes emphasized their positive aspects and high practical value. Negative opinions concerned one basic aspect—stress related to being watched and evaluated. Similar findings were also expressed by Polish medical students in the aforementioned study by Przymuszala et al. [43]. The themes we generated during the analysis reflected, among others, the practical value of our classes and their form of engagement as perceived by students. The authors of a study on pharmacy students’ opinions about their studies in Ireland generated the theme “Learning by Doing,” which was comparable to our “Just like in a real pharmacy” theme [50]. Like the Polish students in our study, Irish students emphasized the significant practical usefulness of active teaching methods, which allow for the acquisition of practical skills and are more engaging than passive learning. This leads us to believe that classes involving active teaching methods are crucial for preparing students to enter a professional role. Research among South African students showed that after the introduction of an educational intervention, which consisted of additional classes conducted using the active team-based learning (TBL) method, the students’ self-assessment of readiness for the clinical analysis of patient cases increased [51]. Our previous research also showed that students believe that there are not enough practical classes in pharmacy studies, that there is a lack of opportunities to learn how to apply their knowledge practically, and that there are too few classes related to conducting PC [14]. The thematic analysis of opinions after our intervention showed that the teaching methods we used and the issues discussed during classes had the expected practical dimensions.

### Limitations

This pilot study has several limitations. First, the impact of the planned intervention was evaluated among a limited group of students who participated in classes during one academic year and in one location. Future studies should concentrate on confirming our findings with a larger group of students from different locations and varying years of study. Second, there was a lack of a control group in which the same classes were conducted using previous or less advanced teaching methods. Further research in this area should strive to compare the impact of less advanced teaching methods on students’ self-efficacy. Another limitation was the absence of a structured exam that could evaluate the knowledge and skills acquired during the course. This was due to time constraints and the fact that the classes during which our surveys were conducted were only a part of the whole subject. Finally, we believe that it would be beneficial to the study if the role of the student in the role-playing was indicated, which could allow for the assessment of the influence of the students’ roles during classes on the examined parameters.

## Conclusions

The educational methods used in our study were found to be effective and to increase students’ self-confidence and self-efficacy. Thus, in our opinion, this approach may be incorporated into an updated pharmacy curriculum. Conducting peer role-playing classes in a simulation environment with a debriefing session does not require increased costs. However, although students’ opinions and subjective indicators showed that the presented intervention was effective and well-received, there are limited data on practical comparisons between different teaching methods, and further research in this area is needed. Another important aspect is the impact of the student’s role in a peer role-play on the learning outcomes achieved. Therefore, further in-depth research is needed to fully confirm the effectiveness of simulation exercises in teaching pharmaceutical care and correctly selecting the teaching method for the assumed learning outcomes.

### Electronic supplementary material

Below is the link to the electronic supplementary material.

**Supplementary Material 1:** Pre-post self-assessment questionnaires

## Data Availability

The datasets used and/or analyzed during the current study are available from the corresponding author upon reasonable request. For the purpose of open access, the author has applied a CC-BY public copyright license to any Author Accepted Manuscript (AAM) version arising from this submission.
