# Supplementary material for: Perception of Polish pharmacy students on simulation exercise in pharmaceutical care for diabetes—a pilot study
Source: BMC Med Educ. 2024 Mar 14;24:283. doi: 10.1186/s12909-024-05245-0 (PMC10941357; doi:10.1186/s12909-024-05245-0)
Supplement: Supplementary file 1 — Supplementary Material 1: Pre-post self-assessment questionnaires [file 12909_2024_5245_MOESM1_ESM.docx]

SELF-ASSESSMENT SURVEY – CLASSES AT CSM

**PSEUDONIM**

| Sex:   - Woman - Man | Age (years):  ……………………. | Year of study:  ………………………… |
| --- | --- | --- |
| Are you also a student or graduate of a field other than pharmacy?   - yes - which one? ............................................................. ............................................................. ............................ - NO | | |
| Have you previously participated in medical simulation classes?   - yes - in which ones? ............................................................. ............................................................. ............................ - NO | | |
| Have you previously attended classes on pharmaceutical care in …………………………… ……. .?   - yes - in which ones? ............................................................. ............................................................. ............................ - NO | | |

**1. Self-perceived confidence regarding skills**

**Please read the following statements carefully and mark in the appropriate box to what extent they are consistent with your beliefs about your own competences in providing patient care.**

| STATEMENT | I completely disagree | I disagree to a large extent | I tend to disagree | I have a neutral attitude | I tend to agree | I agree to a large extent | I totally agree |
| --- | --- | --- | --- | --- | --- | --- | --- |
| 1. I am confident that I can communicate effectively with the patient. |  |  |  |  |  |  |  |
| 2. I am convinced that I can provide the patient with knowledge in an understandable way and instruct him in skills (e.g. how to use self-monitoring devices) |  |  |  |  |  |  |  |
| 3. I have appropriate interpersonal communication competences to conduct patient education |  |  |  |  |  |  |  |
| 4. I know how to read and interpret non-verbal messages in a conversation with a patient |  |  |  |  |  |  |  |
| 5. I can identify the patient's health **problems** and respond to them |  |  |  |  |  |  |  |
| 6. I can identify the patient's health **needs** and respond to them |  |  |  |  |  |  |  |
| 7.I have adequate knowledge to educate patients on aspects related to treatment and follow-up …………………… ……. |  |  |  |  |  |  |  |

**2. Self-perceived confidence regarding competencies**

**Please read the following statements carefully and mark in the appropriate box how you rate your skills in particular aspects.**

| STATEMENT | Very difficult | Difficult | Rather difficult | Neither difficult nor easy | Rather easy | Easy | Very easy |
| --- | --- | --- | --- | --- | --- | --- | --- |
| 1. Providing the patient with knowledge and instructing him in specific skills is… |  |  |  |  |  |  |  |
| 2. Interpersonal communication is... |  |  |  |  |  |  |  |
| 3.Reading non-verbal messages is… |  |  |  |  |  |  |  |
| 4.Identification and response to patient **problems** is… |  |  |  |  |  |  |  |
| 5. Identifying and responding to patient **needs** is… |  |  |  |  |  |  |  |
| 6. Conducting patient education in the field of treatment and control ……………………………… is… |  |  |  |  |  |  |  |

**3. SELF -EFFICACY is the belief in one's own abilities to achieve a specific goal in various conditions, especially in stressful situations. ( Bandura, Albert. “ Self-Efficacy Mechanism in Human Agency . 1982).**

**Please mark, in the form of a vertical line on the scale below, the place between the two extreme statements, indicating your level of self-efficacy in relation to your ability to communicate with the patient.**

The highest possible confidence in communicating with the patient

Lack of self-confidence in communicating with the patient
